# Diagnostic accuracy of endoscopic ultrasonographic shear wave elastography for assessing early chronic pancreatitis using the Japanese diagnostic criteria 2019

**DOI:** 10.1002/deo2.387

**Published:** 2024-06-11

**Authors:** Shuhei Shintani, Osamu Inatomi, Takuya Okamoto, Kosuke Hiroe, Takaaki Eguchi, Yuki Tomozawa, Akitoshi Inoue, Hidenori Kimura, Atsushi Nishida, Yoshihisa Tsuji, Yoshiyuki Watanabe, Akira Andoh

**Affiliations:** ^1^ Department of Medicine Division of Gastroenterology Shiga University of Medical Science Shiga Japan; ^2^ Department of General Medicine Shiga University of Medical Science Shiga Japan; ^3^ Department of Radiology Shiga University of Medical Science Shiga Japan; ^4^ Department of Endoscopy Shiga University of Medical Science Shiga Japan

**Keywords:** acute pancreatitis, pancreatic fibrosis, propensity score, regression analysis, shear wave elastography

## Abstract

**Background and Aim:**

Endoscopic ultrasound shear wave elastography (EUS‐SWE) can facilitate an objective evaluation of pancreatic fibrosis. Although it is primarily applied in evaluating chronic pancreatitis, its efficacy in assessing early chronic pancreatitis (ECP) remains underinvestigated. This study evaluated the diagnostic accuracy of EUS‐SWE for assessing ECP diagnosed using the Japanese diagnostic criteria 2019.

**Methods:**

In total, 657 patients underwent EUS‐SWE. Propensity score matching was used, and the participants were classified into the ECP and normal groups. ECP was diagnosed using the Japanese diagnostic criteria 2019. Pancreatic stiffness was assessed based on velocity (Vs) on EUS‐SWE, and the optimal Vs cutoff value for ECP diagnosis was determined. A practical shear wave Vs value of ≥50% was considered significant.

**Results:**

Each group included 22 patients. The ECP group had higher pancreatic stiffness than the normal group (2.31 ± 0.67 m/s vs. 1.59 ± 0.40 m/s, *p* < 0.001). The Vs cutoff value for the diagnostic accuracy of ECP, as determined using the receiver operating characteristic curve, was 2.24m/s, with an area under the curve of 0.82 (95% confidence interval: 0.69–0.94). A high Vs was strongly correlated with the number of EUS findings (rs = 0.626, *p* < 0.001). Multiple regression analysis revealed that a history of acute pancreatitis and ≥2 EUS findings were independent predictors of a high Vs.

**Conclusions:**

There is a strong correlation between EUS‐SWE findings and the Japanese diagnostic criteria 2019 for ECP. Hence, EUS‐SWE can be an objective and invaluable diagnostic tool for ECP diagnosis.

## INTRODUCTION

Chronic pancreatitis is characterized by persistent inflammation leading to fibrosis in the pancreatic parenchyma.^1^ Fibrosis induces functional disorders associated with pancreatic endocrine and exocrine dysfunction and adversely affects prognosis. Hence, it is a risk factor for pancreatic cancer.^1^, ^2^, ^3^, ^4^ As fibrosis becomes histologically irreversible with the progression of chronic pancreatitis, early diagnostic approaches have gained attention.^5^ In 2009, the diagnostic criteria for early chronic pancreatitis (ECP) were established in Japan and recognized worldwide.^6^ In 2016, Whitcomb et al. proposed the mechanistic definition concept, indicating that ECP precedes progression to chronic pancreatitis and can be reversed with medical intervention.^3^

Chronic pancreatitis is diagnosed using magnetic resonance cholangiopancreatography, endoscopic retrograde cholangiopancreatography, or endoscopic ultrasound (EUS). Three‐tesla magnetic resonance cholangiopancreatography can noninvasively identify the detailed pancreatic duct morphology.^7^ However, it cannot sufficiently provide details for evaluating the pancreatic parenchyma because of its limited spatial resolution. Endoscopic retrograde cholangiopancreatography is inappropriate for patients without symptoms due to its high invasiveness. By contrast, EUS, based on the Rosemont classification, is a non‐invasive procedure that can provide detailed insights about the pancreatic parenchyma and duct. However, since EUS is operator‐dependent, diagnostic interobserver variabilities may occur.^8^ Thus, a more objective diagnostic tool should be developed.

EUS elastography is a diagnostic modality that quantitatively measures tissue stiffness.^9^ Based on the physical quantity measured, EUS elastography is divided into two measurement methods, which are as follows: strain elastography, which measures tissue distortion in the region of interest (ROI) using the acoustic radiation force impulse, and shear wave elastography (SWE), which measures the velocity (Vs) of shear waves induced by applying a push pulse to the tissue. EUS‐strain elastography assesses the relative stiffness value within the ROI. Moreover, it provides a more objective evaluation by determining the absolute stiffness value within the ROI.^10^, ^11^, ^12^ EUS‐SWE findings strongly correlate with fibrosis stage 12 in diagnosing chronic pancreatitis.

However, the efficacy of EUS‐SWE in diagnosing ECP, characterized by relatively mild fibrosis, has not been fully elucidated. This study aimed to investigate the effectiveness of EUS‐SWE based on the Rosemont classification for confirming ECP diagnosis. Further, the correlation between EUS‐SWE findings and clinical features was explored.

## METHODS

### Characteristics of the patients and definition of ECP

The Shiga University of Medical Science Ethics Committee approved this retrospective study (approval no.: R2021‐143). All patients provided written informed consent before undergoing EUS. EUS procedures were retrospectively performed from April 2020 to December 2021 at a single institution. Subsequently, the medical records of the patients were reviewed. Exclusion criteria included patients with chronic pancreatitis, pancreatic tumors, and any pancreatic abnormality identified in available imaging examinations. Following patient exclusion, data on comorbidities, laboratory examination results, alcohol consumption, and smoking history were investigated. EUS procedures were performed by two or more endoscopists (who performed >1000 EUS examinations). The current study included patients diagnosed with ECP based on the Japanese diagnostic criteria 2019 (ECP group)^13^ and healthy individuals without pancreatic abnormalities (normal group). In this study, EUS results were used as inclusion criteria. The ECP group had at least two of the following EUS findings: 1) hyperechoic foci (non‐shadowing) or strands, 2) lobularity, 3) hyperechoic main pancreatic duct (MPD) margin, and 4) dilated branch ducts.^13^ B‐mode imaging was conducted to assess the pancreatic parenchyma and pancreatic ducts. The sizes of each finding were as follows: hyperechoic foci (non‐shadowing) or strands, ≥3 mm; lobularity, ≥5 mm; and dilated side branches, ≥1 mm. The presence of three or more hyperechoic foci/strands was considered positive. A positive hyperechoic MPD margin was defined as a distinct structure found in more than half of the main duct in the pancreatic body.^13^ To diagnose ECP, two endoscopists blinded to clinical information independently evaluated EUS imaging findings to determine their presence or absence. For the disagreed results, they were determined by consensus. In addition, patients with definite and possible ECP accompanied by the following three and two clinical features were assigned to the ECP group. The following clinical features in the Japanese diagnostic criteria 2019 were used to identify patients with ECP: (1) recurrent upper abdominal or back pain, (2) high serum and urinary pancreatic enzyme levels, (3) abnormal pancreatic exocrine function, (4) continuous heavy alcohol consumption (≥60 g/day of pure ethanol) or mutation in the pancreatitis‐associated genes, and (5) a previous history of acute pancreatitis. The normal group comprised individuals without symptoms of abdominal or back pain, a history of acute or chronic pancreatitis, and evidence of pancreatic tumors or stenosis/dilation of the pancreatic duct. The pancreatic function was evaluated using the urinary excretion rate of *N*‐benzoyl‐L‐tyrosyl‐p‐aminobenzoic acid (PFD Oral Solution; Eisai), and the lower limit of pancreatic function based on the pancreatic functional test was 70%.

### EUS‐SWE and screening procedure

**FIGURE 1:**
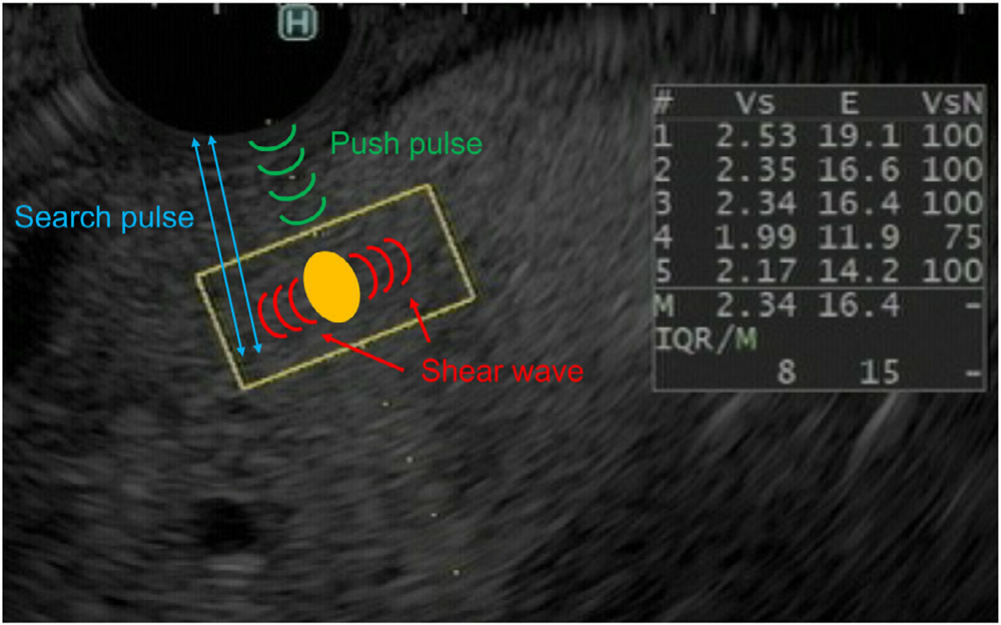
The mechanism of endoscopic ultrasound shear wave elastography (EUS‐SWE). A shear wave is generated by applying a push pulse from the probe to the target organ. The hardness of the organ is estimated by measuring the velocity of the shear wave (Vs, m/s) with a search pulse. The percentage of the net amount of effective shear wave velocity (VsN, %), which indicates the reliability of the Vs, is measured simultaneously.

All EUS procedures were performed using a gastrointestinal endoscope (GF‐UCT260; Olympus) and an ultrasound device (ARIETTA850, FUJIFILM Medical Systems). All examinations were conducted under sedation with midazolam and pentazocine, and propofol was additionally used if regular sedation was insufficient. EUS and EUS‐SWE were performed during the same examination day. EUS‐SWE parameters were obtained immediately after visualizing the pancreatic body using B‐mode imaging (Figure 1). The pancreatic parenchyma, avoiding vessels, main duct, and cysts were measured by placing a 10 × 5‐mm ROI, and the measurements were performed at least five times using a method described in a previous study.^14^ The net amount of effective shear wave Vs, a reliability index of Vs, was >50%, concerning previous reports on SWE using abdominal ultrasonography^.^ ^15^, ^16^ The Vs was evaluated based on the median of all target values, and the interquartile range (IQR) was calculated.

### Statistical analysis

Propensity score matching was performed after adjusting for age and sex to decrease patient selection bias, considering reports that age affects pancreatic stiffness within the pancreas. For non‐parametric analysis, the Mann–Whitney U test was used to assess continuous variables, and the Fisher's exact test or the chi‐square test was utilized to evaluate nominal variables. A p‐value of <0.05 was considered statistically significant. As the diagnostic criteria for ECP consist of subjective EUS imaging findings, interobserver agreement was analyzed using Cohen kappa value and categorized as poor (<0.20), fair (0.21–0.40), moderate (0.41–0.60), good (0.61–0.80), and very good (0.81–1.00). The correlation between the number of EUS criteria and the Vs values on EUS‐SWE was evaluated using Spearman's rank correlation coefficient. The diagnostic performance of ECP based on the Rosemont criteria, which was used as the reference standard, was assessed by calculating the area under the curve (AUC) using ROC, and the cutoff value was determined. All statistical analyses were performed using EZR version 1.40 (Saitama Medical Center, Jichi Medical University).

## RESULTS

### Patient backgrounds

**FIGURE 2:**
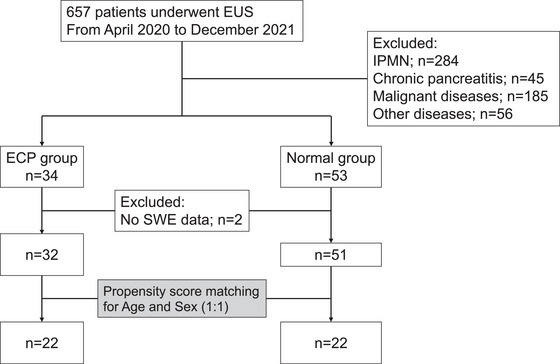
Flow diagram. In total, 657 patients underwent endoscopic ultrasound (EUS). We excluded 284 patients with intrapapillary mucinous neoplasm, 45 with chronic pancreatitis, 185 with malignant disease, 56 with other diseases, and two with insufficient data on EUS‐shear wave elastography findings. Finally, 34 patients with early chronic pancreatitis and 53 without pancreatic diseases were evaluated. After propensity score matching, 22 patients from each group were included in the analysis.

In total, 657 patients underwent EUS procedures. Patients with ECP (*n* = 34) and individuals without any pancreatic diseases (*n* = 53) were identified after excluding patients with intrapapillary mucinous neoplasm (*n* = 284), chronic pancreatitis (*n* = 45), malignancy of the biliary and pancreas (*n* = 185), and others (*n* = 56). Two patients with insufficient data were excluded from the ECP and normal groups (Figure 2). Finally, 32 patients with ECP and 51 individuals without pancreatic diseases were enrolled in this study. After propensity score matching, 22 patients in the ECP group (mean age: 66.8 years, men: 14) and 22 in the normal group (mean age: 66.8 years, men: 14) were included in the analysis. The comorbidities in the ECP and normal groups included hypertension (10 [45.5%] vs. 7 [32.3%]), dyslipidemia (7 [31.8%] vs 9 [40.9%]), and type 2 diabetes (4 [18.1%] vs. 5 [22.7%]). Heavy drinkers were more frequent in ECP than the normal group (8 [36.4%] vs. 2 [9.1%]), whereas smoking history was equally seen in both groups (8 [36.4%] vs. 7 [31.8%]). The ECP group presented with clinical features related to ECP diagnosis, which included abdominal and back pain (*n* = 20, 90.9%), abnormal pancreatic enzyme levels (*n* = 20, 90.9%), and a history of acute pancreatitis (*n* = 4, 18.2%). However, the normal group did not observe these features (Table 1). Five patients (31.3%) presented with decreased pancreatic function in the patients examined with pancreatic functional tests.

**TABLE 1:** Characteristics of the patients.

	Normal group (*n* = 22)	ECP group (*n* = 22)	*p*‐value
Age, ±SD	66.8 (±9.1)	66.4 (±8.8)	0.90
Male sex, *n* (%)	14 (63.6)	14 (63.6)	1
BMI, ±SD	22.5 (±2.7)	23.0 (±4.0)	0.63
Comorbidities, *n* (%)			
Hypertension	7 (32.2)	10 (45.5)	0.54
Dyslipidemia	9 (40.9)	7 (31.8)	0.76
Diabetes mellitus	5 (22.7)	4 (18.1)	1
Heavy alcohol consumption, *n* (%)	2 (9.1)	8 (36.4)	0.07
Smoking, *n* (%)	7 (31.8)	8 (36.4)	1
Clinical symptoms, *n* (%)			
Abdominal or backpain	0	20 (90.9)	<0.001
Abnormal pancreatic enzyme	0	20 (90.9)	<0.001
History of pancreatitis	0	4 (18.2)	0.11
Decreased pancreatic function based on the PFT^*^	‐	5 (31.3)	NA

Abbreviations: BMI, body mass index; ECP, early chronic pancreatitis; NA, not available; PFT, pancreatic function test..

* Sixteen patients in the ECP group had available data.

### EUS findings and EUS‐SWE

**FIGURE 3:**
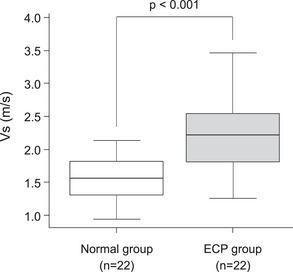
Comparison of velocity (Vs) values between the early chronic pancreatitis (ECP) and normal groups. The Vs values of the ECP group were significantly higher than the normal group's (*p* < 0.001).

**FIGURE 4:**
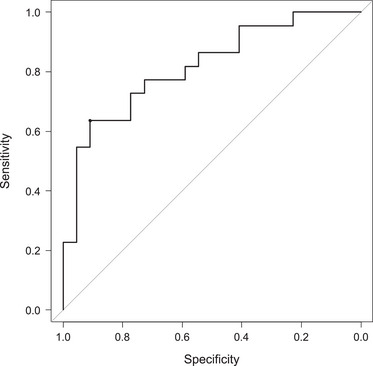
Receiver operating characteristic curves (ROCs) of the velocity (Vs) values for early chronic pancreatitis. The ROC analysis revealed that the area under the curve (AUC) was 0.82 (95% confidence interval: 0.69–0.94). The Vs cutoff value for the diagnostic accuracy of ECP was 2.24 m/s.

**FIGURE 5:**
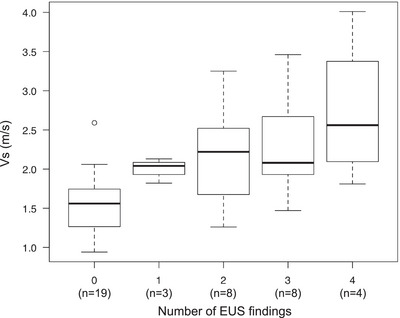
Correlation coefficient between velocity (Vs) values and the number of endoscopic ultrasound (EUS) findings. The Vs values and the total number of EUS findings were significantly correlated (rs = 0.626, *p* < 0.001).

The overall kappa values (95% confidence interval [CI]) of interobserver reliability (IOR) for each EUS finding were as follows: hyperechoic foci and strands: 0.95 (0.86–1.0), lobularity: 0.67 (0.40–0.94), hyperechoic main duct margin: 0.68 (0.43–0.92), dilated branch ducts: 0.56 (0.27–0.86). Table 2 shows the EUS findings. In total, 22 (100%) patients in the ECP group presented with hyperechoic foci and strands. The ECP group was more likely to present with lobularity, hyperechoic duct wall, and dilated side branches than the control group, with incidence rates of 12 (54.5%), 15 (68.2%), and 11 (50.0%), respectively (*p* < 0.001). Three (13.6%) patients in the normal group exhibited hyperechoic foci and strands. Figure 3 shows the EUS‐SWE results. The ECP group had significantly higher Vs values than the normal group (2.31 ± 0.67 [IQR: 1.36–2.93] m/s vs. 1.59 ± 0.40 [IQR: 1.41–2.03] m/s; *p* < 0.001). The success rate of Vs measurement was 98.7% (228 of 231 sessions). The cutoff Vs values for the diagnostic accuracy of ECP was 2.24 m/s (AUC: 0.82; 95% CI, 0.69–0.94) (Figure 4). A strong correlation was observed between high Vs values and the total EUS findings (rs = 0.626, *p* < 0.001, Figure 5). There was no significant difference in the Vs values based on the presence or absence of pancreatic insufficiency (2.09 ± 0.44 vs 2.21 ± 0.77, *p* = 0.68). Multiple regression analysis revealed that a history of acute pancreatitis and the presence of ≥2 EUS findings were independent factors of high Vs values (Table 3)

**TABLE 2:** Endoscopic ultrasound findings.

	Normal group (*n* = 22)	ECP group (*n* = 22)	*p*‐value
EUS findings			
Hyperechoic foci and strands	3 (13.6)	22 (100)	<0.001
Lobularity	0	12 (54.5)	<0.001
Hyperechoic duct wall	0	15 (68.2)	<0.001
Dilated side branch	0	11 (50.0)	<0.001

Abbreviations: ECP, early chronic pancreatitis; EUS, endoscopic ultrasonography.

**TABLE 3:** Multivariate linear regression analysis of increased pancreatic stiffness on endoscopic ultrasound shear wave elastography.

	Regression coefficient (95% CI)	Standard coefficient	*p*‐value
Heavy alcohol consumption	0.144	0.226	0.53
History of acute pancreatitis	0.631	0.309	0.04
Abdominal pain or backpain	–0.494	0.442	0.27
Endoscopic findings (≥2 criteria)	1.005	0.451	0.03

## DISCUSSION

Our study revealed that patients with ECP had significantly higher pancreatic stiffness than those without pancreatic diseases. This is the first report that quantitatively evaluated pancreatic stiffness on EUS‐SWE in patients with ECP. Based on our findings, EUS‐SWE can be used to objectively diagnose advanced fibrosis in chronic pancreatitis and ECP with mild fibrosis. Further, a history of acute pancreatitis and multiple endoscopic ultrasound findings were found to be independent factors of increased pancreatic stiffness.

ECP is a precursor to chronic pancreatitis and a reversible condition based on the Japanese diagnostic criteria 2019. Conventional imaging modalities such as MRI and endoscopic retrograde cholangiopancreatography cannot sufficiently identify minor morphological changes in the pancreatic parenchyma on ECP. Meanwhile, EUS is recommended in the Japanese diagnostic criteria 2019. The endoscopic findings on EUS comprise two factors reflecting fibrosis in the pancreatic parenchyma (hyperechoic foci and lobularity) and two factors indicating changes in the pancreatic duct (hyperechoic MPD margin and dilated side branches). Still, the EUS findings are subjective and associated with limitations in terms of reproducibility. Hyperechoic MPD margins (κ = 0.771) and hyperechoic foci (κ = 0.741) demonstrate relatively consistent inter‐observer agreements; however, lower in lobularity (κ = 0.706) and dilated side branches (κ = 0.552), and all of them decreases further among non‐expert operators (κ = 0.695, 0.430) (1).^17^ Our data showed similar trends, interobserver agreements for lobularity (κ = 0.67), and dilated side branches (κ = 0.56). The main duct margin (κ = 0.68) was also lower than hyperechoic foci and strands (κ = 0.95). Although differentiating between strands and lobularity, which indicate continuous or regional findings, is crucial, the current diagnostic criteria have limitations in their objectivity.

A more objective and quantitative diagnostic modality is required to evaluate the pancreatic parenchyma in ECP diagnosis to resolve these issues.

EUS‐SWE became available in 2017 with an endoscopic ultrasound scope and high‐definition ultrasound equipment. It is clinically applied as a novel noninvasive diagnostic modality. Ohno et al. reported that EUS‐SWE can measure pancreatic stiffness in various pancreatic diseases with a high success rate of 96.3%–98.8%.^18^ Yamashita et al. have shown that the high diagnostic ability of EUS‐SWE based on the Rosemont criteria, which is the gold standard for chronic pancreatitis, is questioned (cutoff value: 2.1 m/s, AUC: 0.97).^10^ Kuwahara et al. reported the diagnostic ability of SWE on abdominal ultrasonography in chronic pancreatitis, with an AUC of 0.77.^19^ Based on these reports, EUS‐SWE is superior to transabdominal ultrasound for diagnosing pancreatic disease.

Yamashita et al. defined the stages of chronic pancreatitis into four groups, and the indeterminate CP group corresponds to the ECP group in our study. The indeterminate CP group and the normal group were not directly compared. However, the indeterminate CP group was more likely to have higher Vs values than the normal group (1.80 vs. 1.52 m/s).^10^ Similar to our results, this finding suggests that a high pancreatic stiffness can occur continuously from the early stages of chronic pancreatitis before definitive chronic pancreatitis. The primary challenge in EUS‐SWE measurement is the reproducibility of the measured Vs values. Due to measurement errors in Vs value determination, multiple measurements are recommended, and an evaluation based on the median value should be performed. In this study, the IQR deviation was 0.51 for all measurements (ECP group: 0.79, control group: 0.31). Previous research showed variabilities in Vs values on EUS‐SWE. That is, the IQR of Vs of the pancreatic body ranged from 1.51 to 2.53 m/s. The IQR deviation was 0.51, which is consistent with our study. However, this represents measurement results for all pancreatic diseases. This study reported measurements specific to ECP. Nevertheless, more accurate measurement methods should be established in the future.

ECP is characterized by five clinical symptoms, with an average of 2.66. For example, recurrent abdominal pain and back pain are observed in 88.8% of cases. However, exocrine pancreatic insufficiency is less common, with an incidence rate of 13.9%.^7^ Previous studies have shown a correlation between Vs values and pancreatic exocrine and endocrine dysfunction in chronic pancreatitis.^20^ However, our study did not find a significant correlation between Vs values and diabetes mellitus or exocrine dysfunction. This may be attributed to the mild progression of fibrosis in ECP, resulting in a lower incidence of diabetes mellitus and exocrine dysfunction and the presence of other factors beyond pancreatic parenchymal impairment.

Furthermore, our study observed abdominal pain in 90.9% of cases. However, no significant correlation was found between abdominal pain and Vs values. Previous studies found no correlation between pancreatic parenchymal fibrosis and pain in chronic pancreatitis.^21^, ^22^ Therefore, although EUS‐SWE can detect mild fibrosis, it may not adequately reflect the clinical symptoms of ECP.

Multivariate analysis revealed that a history of acute pancreatitis and multiple positive EUS findings were associated with high Vs values in ECP. Repeated acute pancreatitis was more likely to progress to chronic pancreatitis. Hence, pancreatic parenchymal fibrosis may progress more quickly in patients with ECP who have a history of acute pancreatitis compared with those without any history. In addition, a positive correlation between the number of EUS findings and Vs values indicates that weighting based on the number of EUS findings can be considered for fibrosis evaluation.

This study had several limitations. First, it was a retrospective and a single‐center study with a limited number of cases. Second, the Rosemont criteria, which cannot support histological findings, is the gold standard for ECP diagnosis in this study. In general, a definitive diagnosis is made by obtaining tissue samples via EUS‐fine‐needle aspiration for pancreatic cancer and neuroendocrine tumors.^23^ However, obtaining pancreatic parenchymal tissues for chronic pancreatitis diagnosis is not shared except in rare cases such as autoimmune pancreatitis and mass‐forming pancreatitis. Varadarajulu et al. and Trinkudanathan et al. have reported that EUS findings can accurately represent the histological findings of ECP without calcification, and they are not correlated with the degree of fibrosis.^24^, ^25^ Although the correlations between SWE and histological findings are limited to reports on transabdominal SWE, Vs values are considered to be correlated with histological findings and the degree of fibrosis.^26^, ^27^ Therefore, we currently consider EUS‐SWE to be positioned as an adjunctive tool for diagnosing early chronic pancreatitis in relation to the Japanese Diagnostic Criteria 2019. Third, although propensity score matching was performed to adjust for background factors and decrease selection bias, future multicenter studies with larger cases must be performed for further investigation. In conclusion, there was a strong correlation between EUS‐SWE findings and the Japanese diagnostic criteria 2019 for ECP. EUS‐SWE can be an objective and valuable tool for ECP diagnosis.

## CONFLICT OF INTEREST STATEMENT

None.

## ETHICS STATEMENT

The current study was conducted in accordance with the Declaration of Helsinki, was approved by the ethics committee of Shiga University of Medical Sciences, and conformed to its guidelines (approval no.: R2021‐143).
